# International Survey on the Use of Arginine Vasopressin in the Postoperative Management of Single Ventricle Patients

**DOI:** 10.3389/fped.2021.669055

**Published:** 2021-07-26

**Authors:** Vidya R. Raghavan, Eduardo M. da Cruz, Jon Kaufman, Suzanne Osorio Lujan

**Affiliations:** ^1^Department of Pediatrics, University of Colorado Denver School of Medicine, Aurora, CO, United States; ^2^The Heart Institute, Children's Hospital Colorado, Aurora, CO, United States

**Keywords:** arginine vasopressin, single ventricle, survey, international, pediatric

## Abstract

Management of patients with single ventricle physiology after surgical palliation is challenging. Arginine vasopressin has gained popularity in recent years as a non-catecholamine vasoactive medication due to its unique properties. However, data regarding its use in the pediatric population is limited. Therefore, we designed a survey to explore whether and how clinicians use this medication in intensive care units for the postoperative management of single ventricle patients. This international survey aimed to assess usage, practices, and concepts related to arginine vasopressin in pediatric intensive care units worldwide. Directors of pediatric intensive care units who are members of the following international professional societies: European Society of Pediatric Neonatal Intensive Care, Association for European Pediatric and Congenital Cardiology, and Pediatric Cardiac Intensive Care Society were invited to participate in this survey. Of the 62 intensive care unit directors who responded, nearly half use arginine vasopressin in the postoperative management of neonatal single ventricle patients, and 90% also use the drug in subsequent surgical palliation. The primary indications are vasoplegia, hemodynamic instability, and refractory shock, although it is still considered a second-line medication. Conceptual benefits include improved hemodynamics and end-organ perfusion and decreased incidence of low cardiac output syndrome. Those practitioners who do not use arginine vasopressin cite lack of availability, fear of potential adverse effects, unclear indication for use, and lack of evidence suggesting improved outcomes. Both users and non-users described increased myocardial afterload and extreme vasoconstriction as potential disadvantages of the medication. Despite the lack of conclusive data demonstrating enhanced clinical outcomes, our study found arginine vasopressin is used widely in the care of infants and children with single ventricle physiology after the first stage and subsequent palliative surgeries. While many intensive care units use this medication, few had protocols, offering an area for further growth and development.

## Introduction

Infants and children with single ventricle physiology are among the most challenging for providers who care for patients with congenital heart disease. Although these malformations are relatively rare, affecting only 5 of every 100,000 children, their management is complex with patients usually requiring surgical palliation in the neonatal period (1, 2). In the immediate aftermath of surgery, most of these patients suffer from shock states, myocardial depression, and hemodynamic instability. The mainstays of postoperative management include supporting the myocardium and peripheral circulation with vasoactive and inotropic agents (1). Arginine vasopressin (AVP) is a non-catecholamine vasoactive whose use has gained popularity in the last decade due to its unique properties and safety profile. Yet, data is scarce, particularly concerning the single ventricle population.

Compared to other vasoactive agents, AVP offers the unique effects of a vasoconstrictor of venous capacitance that increases systemic vascular resistance without a direct impact on systolic function; nonetheless, it may alter heart rate or myocardial oxygen consumption in some circumstances. Indeed, AVP, a neurohypophyseal hormone secreted by the pituitary gland, acts on V1 receptors and causes smooth muscle-mediated vasoconstriction (3). This is achieved via a mechanism of inhibition of inducible and endogenous nitric oxide synthetase at the cellular level (4). The efficacy with which it causes selective vasoconstriction has led some to recommend AVP for hypoxemia in postoperative Norwood patients as a means of increasing pulmonary blood flow by raising systemic vascular resistance, thus increasing the Qp/Qs (5). As systemic vascular resistance increases under the effect of AVP, caution ought to be exerted with regards to the subsequent increase of the systemic ventricle afterload that may ultimately negatively impact systolic function and stroke volume, notably in the setting of suboptimal preload and systemic ventricle dysfunction. Owing to these effects, AVP is of interest in patients with significant postoperative systemic inflammatory disorders and vasoplegia in whom modulation of the vascular tone is sought. A more recent retrospective study exploring the use of AVP in Norwood patients reported that the initiation of AVP in their postoperative course was temporally associated with improvement in markers of perfusion, including systolic blood pressure, urine output, lactic acid, and pH (6).

Albeit AVP is commonly used in the cardiovascular setting, evidence-based data remains scarce. There is no formal data about its use internationally or the protocols and practices accepted and applied in the cardiac care in intensive care units. The objective of this study was to explore the prevalence, concepts, and methods of AVP use in the postoperative management of pediatric single ventricle patients in intensive care units worldwide.

## Materials and Methods

### Study Design

We performed a cross-sectional international survey aimed at neonatal, pediatric, and cardiac intensive care unit directors regarding their use of AVP. The survey inquired about attitudes regarding safety, indications for use, benefits, advantages, and disadvantages. This study was deemed exempt by the University of Colorado Institutional Review Board.

### Survey Development

We designed an original survey to assess use and concepts regarding AVP in the pediatric intensive care unit after cardiac surgery. The questions were developed by the authors based on their experience in designing international surveys currently published in peer-reviewed journals and their own experience with the use of AVP in the single ventricle patient population in a large university-affiliated free-standing pediatric hospital. The survey was built with a dynamic branching logic format to provide follow-up questions specific to the survey respondents' usage practices (Supplementary Figure 1). The survey was also tested for length and took 7–10 min to complete. All survey questions required a response before proceeding with the subsequent question to ensure comprehensive data gathering.

### Study Subjects

Participants were recruited through the following international societies: European Society of Pediatric Neonatal Intensive Care (ESPNIC), Association for European Pediatric and Congenital Cardiology (AEPC), and Pediatric Cardiac Intensive Care Society (PCICS) directories. The authors utilized a publicly available list to distribute the survey for PCICS; it was distributed with endorsement by AEPC and ESPNIC. The respondents reflect a convenience sample. The survey was provided through a link to Survey Monkey^TM^ conducted between November 2017 and January 2018. Survey responses were collected anonymously, except for the respondents' country of practice.

### Statistical Analysis

Responses for each completed survey were collected and managed using REDCap electronic data capture tools hosted at the University of Colorado REDCap (Research Electronic Data Capture). It is a secure, web-based application designed to support data capture for research studies, providing: (1) an intuitive interface for validated data entry; (2) audit trails for tracking data manipulation and export procedures; (3) automated export procedures for seamless data downloads to standard statistical packages, and (4) procedures for importing data from external sources (7). A descriptive statistical analysis (distribution of responses expressed as percentages) was performed with PRISM and Microsoft Excel (2019). Both fully and partially completed surveys were analyzed and none were excluded. The authors report the percentage (%) for all responses.

## Results

**Figure 1:**
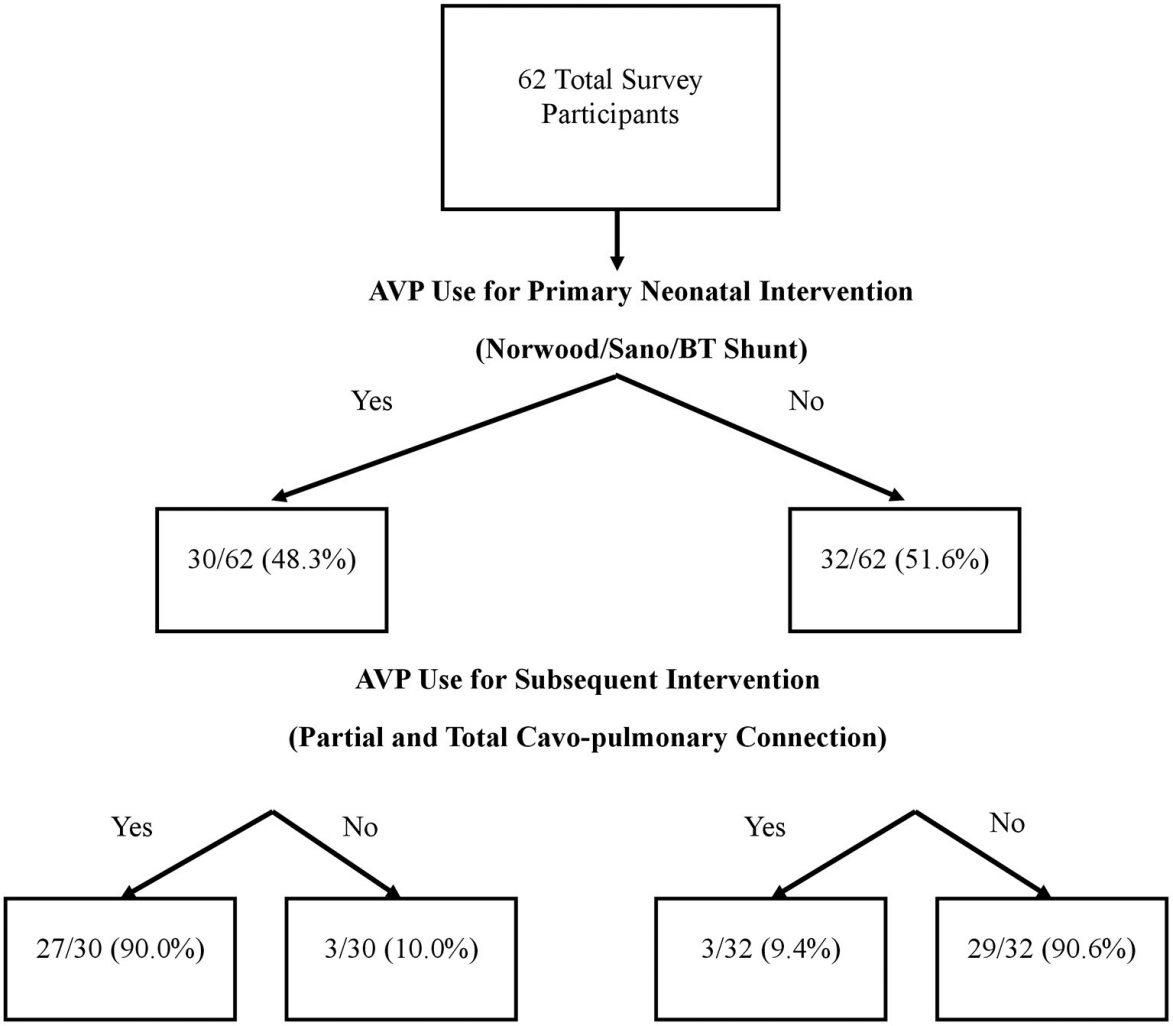
Prevalence of Arginine Vasopressin (AVP) use among survey respondents.

There were 62 survey respondents from 21 different countries spanning five continents (Table 1). Almost half of the respondents (48.4%) use AVP for the postoperative care of neonatal patients undergoing a Norwood operation with a Sano or Blalock-Taussig shunt placement. Among AVP users, the majority (90%) also used AVP for subsequent interventions such as partial or total cavo-pulmonary connections. Interestingly, a small proportion (9.4%) of those who did not use AVP for the neonatal intervention did use it for management in subsequent palliations (Figure 1). Within the group of AVP users, only 27.2% reported standardized practice at their institution, and among them, only one had an institutional protocol for AVP use in these patients.

**Table 1:** Geographic distribution of survey respondents by country and region.

**Region and Country**	**Number of survey participants, *n* (%)**
**Europe**	**29 (46.8%)**
United Kingdom	7
Switzerland	4
France	3
Italy	3
Netherlands	3
Spain	3
Germany	2
Belgium	1
Greece	1
Latvia	1
Portugal	1
**North America**	**25 (40.3%)**
United States	23
Canada	2
**Central and South America**	**4 (6.5%)**
Argentina	1
Colombia	1
Costa Rica	1
Mexico	1
**Asia**	**3 (4.8%)**
Saudi Arabia	1
Singapore	1
United Arab Emirates	1
**Australia**	**1 (1.6%)**
Australia	1
**Total respondents**	**62**

The most frequent indications for AVP use included evidence of vasoplegia or excessive systemic vasodilation (90.6%), refractory shock (68.8%), and hemodynamic instability (75.0%). Respondents also provided the following write-in responses, including “optimize renal perfusion,” “routine post-op management post-Fontan,” and “reduce catecholamine doses if tachyarrhythmias present” (Table 2).

**Table 2:** Arginine Vasopressin (AVP) Practice Patterns and Indications for Use Among Users.

**AVP practice patterns and indications**	**% Among users**
**Consistent practice amongst your colleagues regarding AVP usage**	
Consistent Practice for AVP Usage	27.3%
No Consistent Practice	72.7%
**Frequency of echocardiography prior to AVP administration**	
Echo routinely obtained	15.6%
Echo not routinely obtained	84.4%
Indications for AVP among users^*^	
Evidence of vasoplegia	93.5%
Postoperative hemodynamic instability	77.4%
Refractory Shock	70.9%
Routine postoperative management	6.4%
Routine intraoperative management	3.2%
**AVP utilized as a first or second-line choice**	
First-line	10.0%
Second-line	90.0%
**AVP dosage**	
Less than 0.0003 U/kg/min	12.9%
0.0003–0.0006 U/kg/min	54.8%
0.0006–0.0009 U/kg/min	16.2%
Greater than 0.0009 U/kg/min	9.7%
Other	6.4%
**Relationship between AVP and enteral feeding**	
AVP is a contraindication to enteral feeding	41.9%
AVP is not a contraindication to enteral feeding	58.1%
**Presence of umbilical arterial or venous catheters contraindication to AVP usage**	
Umbilical arterial catheters are a contraindication to AVP	6.5%
Neither are a contraindication for starting AVP	93.5%

(*) *More than one response accepted*.

The majority of AVP users reported 0.0003 to 0.0006 U/kg/min of AVP as the most commonly used dosage (54.8%). A minority of respondents used <0.0003 U/kg/min (16.4%), 0.0006 to 0.0009 U/kg/min (12.9%), or >0.0009 U/kg/min (9.7%) (Table 2).

There was a consensus among users that AVP was regarded as a second-line drug by 90%. Similarly, the presence of an umbilical artery catheter or umbilical vein catheter was not considered a contraindication to AVP use by 93.5%. An echocardiogram was also not routinely obtained before starting AVP according to the majority of respondents, 84.4%. Enteral feeding while receiving AVP was divisive, with 58.1% reporting that they feed, while the remaining 41.9% consider enteral nutrition to be a contraindication (Table 2).

Amongst AVP non-users, the most common reasons for not using the drug included lack of availability, concern about potential adverse effects, absence of clear indication for use, and absence of evidence suggesting improved outcomes (Table 3). Alternate first-line drugs used instead of vasopressin included norepinephrine and phenylephrine (Table 3).

**Table 3:** Reasons for Arginine Vasopressin (AVP) Avoidance and Alternate Medications Used Among Non-Users.

**AVP avoidance and alternate medications**	**% Among non-users**
**Alternative agents to AVP**	
Norepinephrine	85.2%
Phenylephrine	14.8%
Primary reason AVP not used among non-users^*^	
AVP not available	27.2%
Concern about potential adverse events	25.9%
Absence of clear indication for use	22.2%
Absence of evidence suggesting improved	14.8%
outcomes compared to other agents	
Other	7.4%
No response	2.5%

(*) *More than one response accepted*.

**Figure 2:**
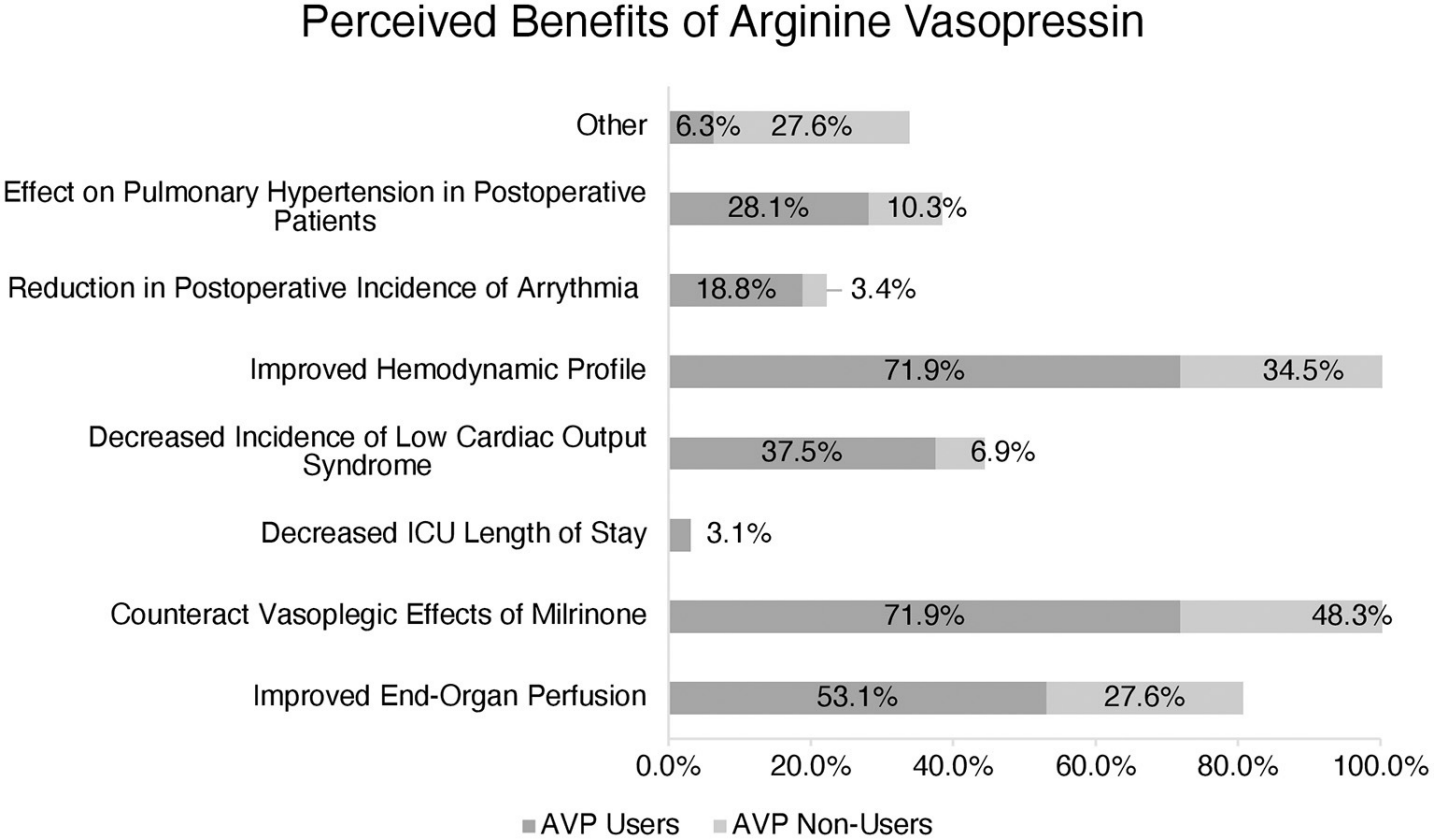
Perception of Arginine Vasopressin (AVP) benefits amongst users vs. non-users. (*) More than one response accepted.

AVP users reported several perceived benefits including improved hemodynamic profile, counteraction of the potential vasodilatory effects of milrinone, improved end-organ perfusion, decreased incidence of low cardiac output syndrome, and impact on pulmonary hypertension in postoperative patients (Figure 2).

**Figure 3:**
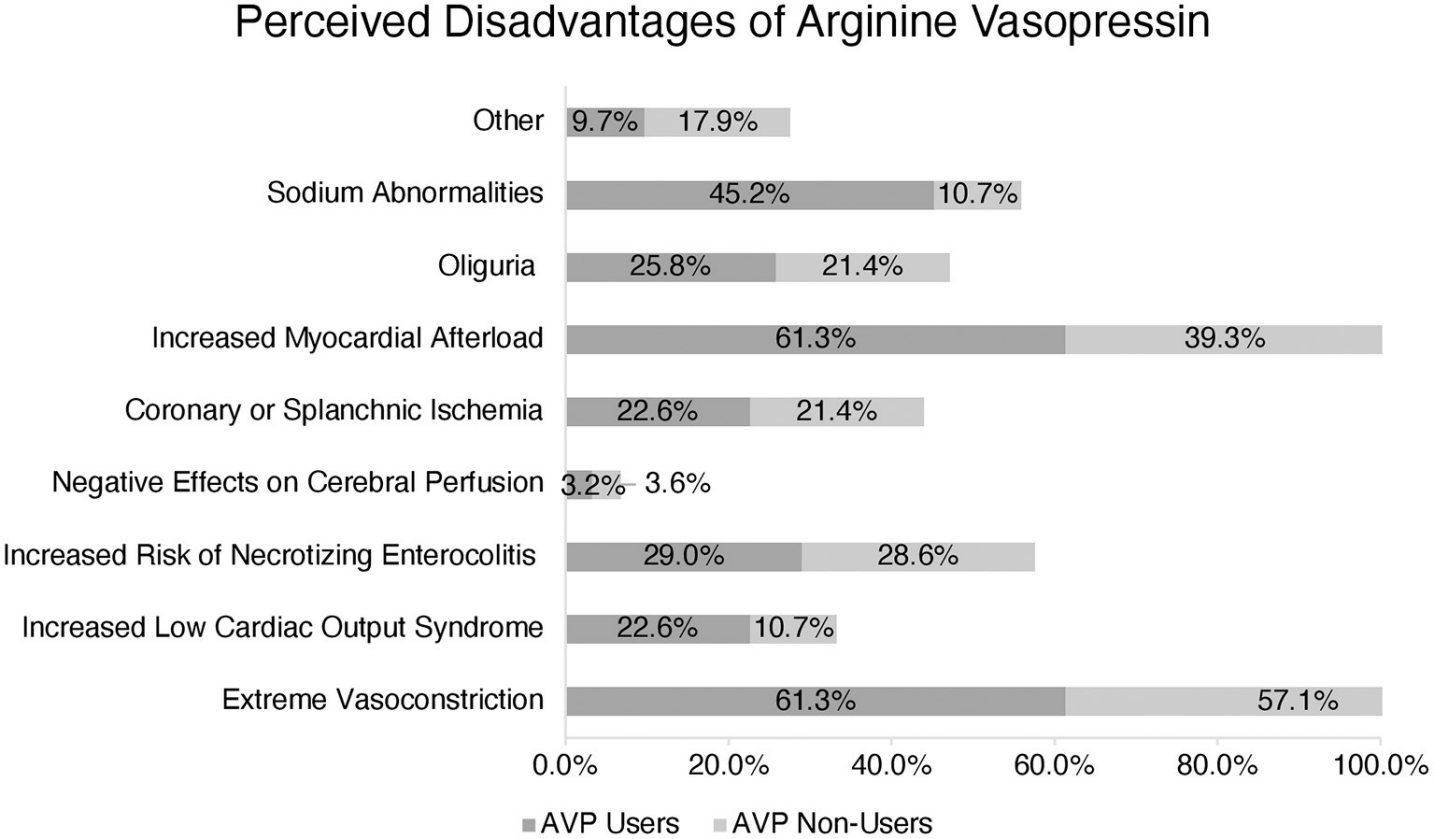
Perception of Arginine Vasopressin (AVP) disadvantages amongst users vs. non-users. (*) More than one response accepted.

The most common described disadvantages cited by both AVP users and non-users included extreme vasoconstriction and increased myocardial afterload: 61.3 and 57.1%, respectively. In a multiple-choice option question, AVP users also reported sodium abnormalities (45.2%), increased risk of necrotizing enterocolitis (29.0%), oliguria (25.8%), increased risk of low cardiac output syndrome (22.6%), and coronary or splanchnic ischemia (22.6%) as other disadvantages. AVP non-users also perceived increased risk of necrotizing enterocolitis, coronary or splanchnic ischemia, and oliguria as disadvantages and reasons not to use this medication (Figure 3).

## Discussion

This study attempts to understand the concepts around the use of the AVP in a population of single ventricle patients for which there is controversy about the virtues or the disadvantages of the drug. This manuscript shares descriptive data about these concepts and attempts to better characterize practices to promote consistency in the future. It provides a valuable insight into practices with AVP in 63 programs across 21 different countries.

In the high-risk and challenging population of postoperative palliation of single ventricle patients, AVP seems to be of interest among pediatric cardiac intensivists for its unique properties. Vasopressin has a wide range of dose-dependent physiologic effects on the cardiovascular system, arguably by increasing vascular tone and improving inotropy. Moreover, it does so without directly increasing myocardial oxygen demand or heart rate, although changes in those parameters may be indirectly induced (3, 5, 6, 8–14). The virtues of the drug are not merely restricted to the effects on the vasculature. V2 and V3 receptors in the renal collecting duct are stimulated by AVP to reabsorb urea, sodium, and water (14, 15). In addition, stimulation of cardiac oxytocin receptors, which have been localized in all cardiac chambers, leads to the production of atrial natriuretic peptide (10). Last but not least, purinergic receptors also bind AVP (8). Indeed, AVP binds to V_1_ receptors on vascular smooth muscle to cause vasoconstriction through the IP_3_ signal transduction pathway and Rho-kinase pathway, which increases arterial pressure. Purinergic receptors are also present in the heart and coronary arteries, although there are conflicting data regarding the activation of these receptors and the subsequent clinical effect (12, 13, 16).

Some reports have shown an absolute or relative deficiency of vasopressin after cardiopulmonary bypass surgery, and the use of exogenous AVP resulted in improvement in the hemodynamic status. These data support AVP use for neonates with vasodilatory or catecholamine-resistant shock after cardiopulmonary bypass (5, 17, 18). This would seem to agree with Alten et al. and with Grant et al., whose studies on the routine use of AVP in the postoperative period reported lower use of fluid resuscitation and catecholamines (6, 19). However, Morrison et al. seemed to contradict these findings by demonstrating that vasopressin levels in pediatric patients after cardiopulmonary bypass are markedly elevated. Additionally, their results suggested that exogenous use of AVP was ineffective or potentially detrimental in its effects on cardiac output and organ perfusion (20). As reflected in our survey, each of these studies note that there is little consensus regarding appropriate dosing, indications for use, risks or benefits, and this lack of consistency has been well-documented (21).

Rosenzweig et al., and Lechner et al., have previously characterized the effect of vasopressin on excessive vasodilation and refractory shock in a population of neonates with catecholamine-resistant vasodilation after cardiopulmonary bypass. In addition, both described improved blood pressure in the patient population that received AVP, which supports this indication (17, 18). Thus, the findings of these studies echo the indications for AVP use cited by survey respondents.

To the best of our knowledge, no studies have determined the appropriate dosage of AVP in pediatric patients. Among the pediatric studies mentioned, dosages have ranged between 0.0003 and 0.002 U/kg/min, primarily extrapolated from adult literature. Lechner et al. reported no ischemic effects with dosages up to 0.001 U/kg/min (17). Our survey determined ranges based on our experiences with AVP in the cardiac intensive care unit at Children's Hospital Colorado. The survey results show that AVP users reported low-dose AVP (0.0003–0.0006 U/kg/min) as the most frequently used dose. Dosages of <0.0003 U/kg/min and >0.0009 U/kg/min were not as commonly reported. We believe this is the first study to describe the most widely used AVP dosage for pediatric single ventricle patients after cardiopulmonary bypass with an international perspective.

The use of umbilical access via arterial or venous catheter was not considered a contraindication to the majority of AVP users. Most providers did not obtain an echocardiogram before the initiation of AVP to document the presence or absence of ventricular dysfunction or atrioventricular or semi-lunar valve regurgitation. This practice could be supported by the results of a retrospective study by Iliopoulos et al., which found that ventricular dysfunction was not a predictor of hemodynamic response to AVP (11). Initiation of enteral feeding while receiving vasopressin was fairly split between respondents. Reasons for avoiding enteral feeding may relate to the severity of the patient's baseline clinical condition rather than to the use of AVP, especially as most AVP users felt AVP was a second-line medication. Porcine models have shown a decrease in blood flow in the gastric mucosa after cardiopulmonary bypass, exacerbated by the use of AVP (9, 22), which could pose a theoretical risk of necrotizing enterocolitis. However, Alten et al. reported no cases of necrotizing enterocolitis and adequate tolerance of enteral feeds in all patients with early initiation of AVP after cardiovascular bypass surgery (19).

AVP non-users reported the preferential use of catecholamines including epinephrine, norepinephrine, alpha-1-adrenergic agonist, and phenylephrine instead of AVP in the setting of a need for the vasoconstrictive effect of vascular beds. These drugs have similar sympathomimetic effects targeting the peripheral vasculature as potent vasoconstrictors. There is also a long history and likely greater comfort with using these agents. Future studies should investigate the possibility of an age or experience bias or association with the use of AVP and the providers who administer it.

Drugs of this nature are an attractive alternative because the challenge of managing these single ventricle patients can be related to the clinical condition of vasoplegia and systemic inflammatory response syndrome as a result of cardiopulmonary bypass (23). Vasoplegia is a form of vasodilatory shock characterized by low systemic vascular resistance with preserved or increased cardiac output (24). The pathophysiology of postoperative vasoplegia in pediatric cardiac patients may be complex and is incompletely understood. It may occur secondary to a profound systemic inflammatory response induced by surgical trauma, exposure of blood to foreign surfaces of the cardiopulmonary bypass circuit, reperfusion injury, and possibly release of endotoxin from bacterial translocation. It may also reflect an absolute or relative deficiency of endogenous vasopressin in children (25, 26). These patients may develop catecholamine-resistant hypotension or extreme vasodilation, and several drugs may be required to treat it adequately but could ultimately be ineffective in managing the hemodynamic state.

The lack of availability of AVP in the pharmaceutical paraphernalia was the most common reason the drug was not utilized by non-users. Yet, in four of the five continents included, there was reported use of AVP, which suggests against specific geographic limitations to availability. The remaining responses reflect the need for further studies regarding the use of vasopressin in the pediatric population, including concern for potential adverse effects, absence of clear indication for use, and absence of evidence suggesting improved outcomes.

The benefits reported by AVP users included improved hemodynamics, counteracting vasoplegia, and improving end-organ perfusion, which echoes the findings of a previous publication from our group (6). However, it is worth noting that AVP non-users also reported these same benefits as AVP users, indicating that other factors influence their preference not to use the drug in the clinical setting.

AVP users and non-users reported extreme vasoconstriction and increased myocardial afterload as potential adverse effects. Indeed, AVP must be used cautiously in the postoperative pediatric patient with evidence of ventricular dysfunction, and the potential for organ or extremity-specific deleterious vasoconstriction needs to be considered. However, it is worth noting that none of the studies in pediatric patients receiving AVP after cardiopulmonary bypass described such adverse effects in their subjects (5, 6, 17–19). While AVP non-users selected necrotizing enterocolitis, oliguria, and splanchnic/coronary ischemia as the most common perceived disadvantages, these were less popular responses among AVP users.

AVP users also reported sodium abnormalities as a disadvantage, but this was not a frequent response from AVP non-users. Two retrospective studies have reported an increased incidence of hyponatremia associated with the patients who received vasopressin post-operatively (6, 27). Further studies should evaluate the effects of AVP on sodium balance in the pediatric cardiac intensive care unit.

Limitations of this study were inherent to the survey nature, which relies on the solicited subjects' willingness to respond and complete the survey and provide accurate responses. The methodology used also reflects a convenience sampling which may lend an inherent bias to the survey respondents. Also, answers may not have reflected the variations in practice within the Directors' programs. Although the survey was sent to the very large general membership of the societies, it is unknown how many Unit Directors were reached, making it impossible to calculate an accurate response rate, in spite of this, 62 responses from 21 countries seems remarkable to the authors. By targeting Unit Directors through the databases of the significant international societies related to the field, the study did not capture those not enlisted in such scholar societies. Although not capturing all practices around the world, this survey provides a unique background of consistencies and discrepancies in the use of AVP, which remains of great importance. A few of the survey respondents did not complete the entirety of the survey; thus, only the information entered could be collected and analyzed. Additionally, it may have been more illustrative to collect further information about the centers where the respondents practice, such as the number of surgeries performed and beds in the intensive care unit, to better contextualize their AVP use. It was not the nature of the study to analyze outcomes of stage 1 single ventricle palliation or correlation of the latter with the use of vasopressin. Last but not least, this survey depicts practices but also individual concepts rather than documented data. Notwithstanding that fact, it does provide for the first time in literature documentation of international practices regarding AVP among single ventricle pediatric patients.

## Conclusions

Even in the absence of robust investigational data to support the routine use of AVP in pediatric patients after cardiac surgery, this survey responses demonstrate that AVP use is relatively widespread in the postoperative management of patients with single ventricle congenital heart disease. It also suggests higher general interest and ubiquity of use than was previously described. Major highlights of this study relate to the fact that approximately half of the surveyed practitioners use AVP to manage the first-stage palliation for single ventricle patients, albeit many consider AVP as a second-line drug. Notwithstanding that fact, the vast majority (90%) of these clinical providers also use AVP in subsequent interventions, which may reflect a higher level of trust in the drug in patients beyond the neonatal period. Of significant importance, we documented that protocols are scarcely used in relation to AVP, which opens avenues toward implementing more consistent practices within the medical community in charge of the postoperative course of single ventricle patients. It is clear that most indications to use AVP in the postoperative period of single ventricle interventions relate to the optimization of tissue perfusion and end-organ function and to antagonize excessive systemic vasodilation secondary to the postoperative inflammatory status allied with side-effects of other cardiovascular drugs. By no means does this survey provide evidence-based data, nor should it influence clinical practices. However, it may spawn clinical studies designed to explore the impacts of AVP on patient outcomes. Nonetheless, a study of this nature may foster further collaboration to implement consistent algorithms for AVP use, gather data collectively, and hopefully better understand the characteristics of this drug and its relation with outcomes in this very complex patient population.

## Data Availability Statement

The raw data supporting the conclusions of this article will be made available by the authors, without undue reservation.

## Ethics Statement

The studies involving human participants were reviewed and approved by University of Colorado School of Medicine Institutional Review Board. The patients/participants provided their written informed consent to participate in this study.

## Author Contributions

VR conceptualized the study and designed the survey along with EC and SO. VR designed the data collection instrument, collected the data, carried out the initial analyses, drafted the initial manuscript, and reviewed and revised the manuscript. SO coordinated and supervised data collection and reviewed and revised the manuscript. JK and EC critically reviewed the survey design and manuscript for intellectual content. All authors approved the final manuscript as submitted and agreed to be accountable for all aspects of the work.

## Author Disclaimer

The contents of the project are the authors' sole responsibility and do not necessarily represent official NIH views.

## Conflict of Interest

The authors declare that the research was conducted in the absence of any commercial or financial relationships that could be construed as a potential conflict of interest.

## Publisher's Note

All claims expressed in this article are solely those of the authors and do not necessarily represent those of their affiliated organizations, or those of the publisher, the editors and the reviewers. Any product that may be evaluated in this article, or claim that may be made by its manufacturer, is not guaranteed or endorsed by the publisher.
