# Supplementary material for: International Survey on the Use of Arginine Vasopressin in the Postoperative Management of Single Ventricle Patients
Source: Front Pediatr. 2021 Jul 26;9:669055. doi: 10.3389/fped.2021.669055 (PMC8350055; doi:10.3389/fped.2021.669055)
Supplement: Supplementary file 1 [file Data_Sheet_1.PDF]

Supplemental Digital Content

Supplemental Figure 1.

1. Select your country (drop down menu of 195 countries)

2. Does your NICU/PICU/CICU utilize AVP in the management of postoperative single ventricle patients (neonatal intervention - Norwood/Sano/BT shunt)?
    a. Yes/No

3. Does your NICU/PICU/CICU utilize AVP in the management of postoperative single ventricle patients (subsequent intervention – partial and total cavo-pulmonary connection)?
    a. Yes/No

4. If yes to either question 2 or 3: Is there consistent practice amongst your colleagues?
    a. Yes/No

5. If yes to question 4: Is the use of AVP driven by a protocol or guideline?
    a. Yes/No

6. If yes to question 5: If your institution has a protocol or guideline for AVP use, what are the criteria to start the drug?
    a. Free text response

7. If yes to question 2 or 3: Is an echocardiogram routinely obtained prior to administering AVP?
    a. Yes/No

8. If yes to question 2 or 3: Identify the most common indication(s) for use of AVP (may choose more than one)
    a. Routine intraoperative management
    b. Routine postoperative management
    c. Postoperative hemodynamic instability
    d. Refractory shock
    e. Evidence of vasoplegia (excessive vasodilation)
    f. Other (please specify) (free text)

9. If yes to question 2 or 3: Is AVP utilized as a first or second line choice?
    a. First/Second

10. If yes to question 2 or 3: What is the usual dosage of AVP used? (if none of these, please pick the value closest)
    a. Less than 0.0003 U/kg/min
    b. 0.0003 – 0.0006 U/kg/min
    c. 0.0006 – 0.0009 U/kg/min
    d. Greater than 0.0009 U/kg/min

    e. Other (please specify)

11. If yes to question 2 or 3: Is the use of AVP a contraindication to enteral feeding?
    a. Yes/No

12. If yes to question 2 or 3: Is the presence of umbilical arterial or venous catheter(s) a contraindication to starting AVP?
    a. Yes/No

13. What are the perceived benefits to the use of AVP? (may choose more than one)
    a. Improved end organ perfusion
    b. Counteract vasoplegia (excessive vasodilation) effect of milrinone
    c. Decrease in low cardiac output syndrome
    d. Decreased length of stay in PICU, NICU or CICU
    e. Improved hemodynamics
    f. Lower incidence of postoperative arrhythmia
    g. Effect on pulmonary HTN in postoperative patients
    h. Other (please specify) (free text)

14. If you do not use AVP for single ventricle patients, what are the alternative drugs used?
    a. Norepinephrine (Noradrenaline)
    b. Phenylephrine
    c. Metaraminol
    d. Not applicable, AVP is used
    e. Other (please specify) (free text)

15. What are perceived disadvantages to the use of AVP? (may choose more than one)
    a. Extreme vasoconstriction
    b. Increased low cardiac output syndrome
    c. Increased risk of necrotizing enterocolitis
    d. Deleterious effect on cerebral perfusion
    e. Coronary or splanchnic ischemia
    f. Increased myocardial afterload
    g. Oliguria
    h. Sodium imbalances
    i. Other (please specify) (free text)

16. If AVP is not commonly used at your institution, what is the primary reason it is not used?
    a. Absence of evidence suggesting improved outcomes
    b. Absence of clear indication for use
    c. Concern about potential adverse effects
    d. Availability
    e. Price
    f. Not applicable, AVP is used

g. Other (please specify) (free text)

17. Please add any other comment that you may deem pertinent
    a. Free text
